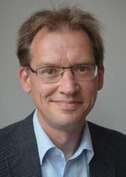# Metal Oxide Nanosheets as 2D Building Blocks for the Design of Novel Materials

**DOI:** 10.1002/chem.201905735

**Published:** 2020-04-01

**Authors:** Melvin A. Timmerman, Rui Xia, Phu T. P. Le, Yang Wang, Johan E. ten Elshof

**Affiliations:** ^1^ MESA+ Institute for Nanotechnology University of Twente P.O. Box 217 7500 AE Enschede The Netherlands

**Keywords:** exfoliation, layered materials, nanomaterials, solution processing, two-dimensional materials

## Abstract

Research into 2‐dimensional materials has soared during the last couple of years. Next to van der Waals type 2D materials such as graphene and h‐BN, less well‐known oxidic 2D equivalents also exist. Most 2D oxide nanosheets are derived from layered metal oxide phases, although few 2D oxide phases can be also made by bottom‐up solution syntheses. Owing to the strong electrostatic interactions within layered metal oxide crystals, a chemical process is usually needed to delaminate them into their 2D constituents. This Review article provides an overview of the synthesis of oxide nanosheets, and methods to assemble them into nanocomposites, mono‐ or multilayer films. In particular, the use of Langmuir–Blodgett methods to form monolayer films over large surface areas, and the emerging use of ink jet printing to form patterned functional films is emphasized. The utilization of nanosheets in various areas of technology, for example, electronics, energy storage and tribology, is illustrated, with special focus on their use as seed layers for epitaxial growth of thin films, and as electrochemically active electrodes for supercapacitors and Li ion batteries.

## Graphical abstract

**Building up**: This Review article provides an overview of the synthesis of oxide nanosheets and methods to assemble them into nanocomposites, mono‐ or multilayer films. In particular, the use of Langmuir–Blodgett methods to form monolayer films over large surface areas, and the emerging use of ink jet printing to form patterned functional films is emphasized. The use of nanosheets in various areas of technology, for example, electronics, energy storage and tribology, is highlighted, with a special focus on their use as seed layers for epitaxial growth of thin films, and as electrochemically active electrodes for supercapacitors and Li ion batteries.

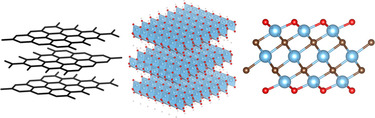

## Introduction

2D materials, or nanosheets, are a class of nanomaterials that draws more and more attention since graphene was discovered.^1^ These 2D materials exhibit a sheet‐like structure, hence the name nanosheets, with lateral dimensions of tens to hundreds of nanometers to even micrometers, and thicknesses no more than 5 nm.^2^ Owing to the effect of spatial confinement in one dimension, nanosheets exhibit a variety of electronic, chemical and optical properties that are not present in their layered bulk counterparts.^3^

A couple of advantages arises from the two‐dimensional nature of nanosheets. Firstly, the electrons are confined in a thin (nanoscale) region.^3^ Their electrons are thus confined to a 2‐dimensional lattice plane, which provides an ideal model system for fundamental studies in condensed matter physics, but also for development of small (opto‐)electronic devices. Since nanosheets have strong in‐plane bonds and the sheet is atomically thin, they tend to show a combination of high mechanical strength, flexibility and optical transparency, which are all highly desirable properties for utilization in various types of devices.^4^ Their atomic thicknesses results in very specific surface areas,^5^ which is a very important property for applications in which the surface area is relevant, such as catalysts and supercapacitors.^6^ Furthermore, the aqueous solution‐based dispersions of nanosheets are suitable precursors for the fabrication of nanosheet‐based films using simple methods like spin‐coating and ink jet printing, usable in applications such as solar cells.^7^ And finally, the fact that all atoms are surface atoms provides a handle to regulate the properties and functionalities of nanosheets by means of surface modification and functionalization, for example with graphene oxide, substitutional element doping, or strain and phase engineering.^8^

Over the last 10 years, we have been synthesizing and using a range of 2D transition metal oxide nanosheets and have been developing methods for their assembly into monolayer films, multilayer heterostructures and hybrid nanocomposites.^9^ This review provides a concise overview of the synthesis and processing of 2D metal oxide nanosheets into functional films, and gives some examples of their application in technological realms as diverse as nanoelectronics,^10^ energy storage^11^ and tribology,^12^ with emphasis on but not limited to research done in our research group. First, the synthesis of oxide nanosheets by top‐down and bottom‐up strategies is summarized. Then, we discuss methods for making morphologically distinct types of nanosheet films by methods such as Langmuir–Blodgett deposition and ink jet printing. In the final sections some examples of functional device components based on or constructed from oxide nanosheets illustrate their wide range of application. In particular, the use of monolayer nanosheets films as seed layers to guide epitaxial growth of functional thin films, for example, ferroelectric, ferromagnetic and memristive oxides, and the application of oxide nanosheets as electrochemically active electrodes for (flexible) supercapacitors and Li ion batteries are emphasized.

## Synthesis of 2‐Dimensional Oxide Nanosheets

### Top‐down synthesis: exfoliation strategies

Various layered materials have been exfoliated into 2D nanosheets. In these layers, the in‐plane atoms are connected to each other via strong chemical bonds, whereas the individual layers stick together by relatively weaker van der Waals interactions.^2^, ^13^ The best‐known example of a layered material is graphite, which consists of a stack of 2D graphene layers that are held together by such weak secondary forces. Other examples of layered materials include hexagonal boron nitride (h‐BN), transition metal dichalcogenides like MoS_2_, MXenes, layered metal oxides and perovskites.^2^ Typically, in these cases the layers are held together by stronger Coulombic and polar interactions.

**Figure 1:**
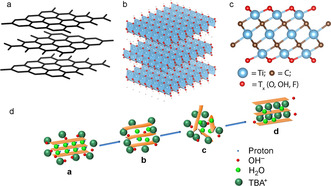
Examples of layered 2D materials: (a) the well‐known graphite structure: three layers of graphene; (b) structure of titanate nanosheets stacked and stabilized by protons; (c) MXene structure of titanium carbide; (d) the proposed intercalation and exfoliation mechanism for layered metal oxides. Reproduced with permission.^14^ Copyright 2015, Wiley‐VCH.

Graphene is the most well‐known 2D material (see Figure 1 a). It is a single atom thick form of graphite. The carbon atoms are arranged in a 2D honeycomb structure (see Figure 1 a), and all of them are covalently bound to three neighbouring carbon atoms through a σ‐bond.^15^ The distance between neighbouring carbon atoms in a graphene nanosheet is 1.42 Å. In non‐exfoliated graphene, that is, graphite, the individual graphene layers are held together by van der Waals forces, where the distance between the layers is approximately 3.35 Å.^16^

Metal trioxides with the general formula MO_3_ (M=Mo, Ta, etc.) are also known to have a layered structure.^2^ For example, MoO_3_ can be thought of as being constructed from distorted MoO_6_ octahedra that share their edges with their neighbouring octahedra, thus forming 2D layers.^17^ The bulk MoO_3_ crystal is essentially a stack of such 2D layers that are held together along the *z*‐axis via van der Waals forces. Other layered oxides that are discussed in more detail below include the layered lepidocrocite‐type compound K_0.8_[Ti_1.73_Li_0.27_]O_4_, the Dion–Jacobson phase KCa_2_Nb_3_O_10_, and K_0.45_MnO_2_. These crystal structures consist of negatively charged 2‐dimensional oxide planes, that is, [Ti_1.73_Li_0.27_]O_4_^0.8−^, Ca_2_Nb_3_O_10_^−^ and MnO_2_^0.45−^, respectively, that are separated by potassium ions located between these planes.

MXenes are 2‐dimensional sheets of transition metal carbides and/or nitrides that can be derived from so‐called MAX phases with the general formula M_*n*+1_AX_*n*_ (*n=*1, 2 or 3). Here M is a transition metal (e.g., Ti, V, Nb), A is an element from group IIIA or IVA (Si, Al, Sn, In), and X is carbon and/or nitrogen.^18^ MAX phases have a layered structure in which the M layers are hexagonally close‐packed and the X atoms fill the octahedral sites.^2^, ^18^ The element A forms 2D planes that separate the M_*n*+1_X_*n*_ layers. The A layer can be etched away selectively from the MAX phase using strong acid solutions, for example HF solution. MXenes sheets with three different structures can thus be formed: M_2_X, M_3_X_2_, or M_4_X_3_. See Figure 1 c for the structure of the MXene titanium carbide.

The layered nature of bulk layered compounds makes them potentially suitable to be exfoliated into their 2D constituents by some top‐down exfoliation process, such as mechanical or ion intercalation exfoliation or one of the methods mentioned below.

The synthesis of 2D nanomaterials with targeted composition, sheet size, layer thickness, crystal phase, defect concentration, and surface chemistry is important for further exploration of their physical and chemical properties, as well as for the development of new applications made using nanosheets as building blocks.^2^ Several synthetic strategies have been developed to make a wide range of nanosheets. These methods include mechanical cleavage, mechanical force‐assisted exfoliation, ion intercalation‐assisted exfoliation, oxidation‐assisted exfoliation, selective etching‐assisted exfoliation, chemical vapour deposition (CVD), and wet‐chemical syntheses.^2^ All methods all fall into one of two categories, namely top‐down and bottom‐up methodologies. CVD and the wet‐chemical syntheses routes for 2D MnO_2_ described in section 2.2 are examples of bottom‐up routes. They are typically based on chemical reactions of specific molecular precursors leading to larger 2D structures. The top‐down routes are limited to the availability of layered parent compounds, but they have the advantage that nanosheets are derived from preformed crystalline parent materials, hence the resulting sheets are also highly crystalline themselves.

The scotch‐tape method to obtain graphene nanosheets from graphite is an example of mechanical exfoliation, but mechanical exfoliation has low yields.^14^ Ultrasonication‐assisted exfoliation can damage the morphology of nanosheets.^19^ On the other hand, ion intercalation exfoliation is normally driven by a chemical reaction and is generally considered to present a much milder synthesis route. More than 40 two‐dimensional compounds made by exfoliating their parent bulk layered compounds have been reported till date. This route can yield large quantities of dispersed nanosheets and is potentially the most promising for large‐scale production processes among the above‐mentioned exfoliation methods.^13^ Considering the exfoliation of layered transition metal oxides using organic quarternary ammonium hydroxides as exfoliation agents (e.g. tetrabutyl ammonium hydroxide, TBAOH), it was generally thought until recently that the structural evolution of the layered parent oxide into the final exfoliated state proceeds via an intermediate “swollen” state in which steric crowding of bulky tetrabutyl ammonium (TBA^+^) ions present between the negatively charged oxide layers initiates the separation of the layers. Such an exfoliation mechanism should involve a relatively slow diffusion‐controlled ion exchange process between bulky TBA^+^ prior to exfoliation.10a However, it has been found that the ion intercalation process is actually driven by a very fast acid–base reaction that can happen even within seconds once the reactants are mixed.^14^

The exfoliation of one of the most well‐known layered metal oxides, lepidocrocite‐type titanate H_1.07_Ti_1.73_O_4_⋅H_2_O (HTO), starts with synthesizing the parent layered bulk compound K_0.8_Ti_1.73_Li_0.27_O_4_ (KLTO). KLTO is made by solid state synthesis in an oven (see ref. 14). The potassium ions reside between the Ti_1.73_Li_0.27_O_4_ layers, to charge‐stabilize the layered bulk compound. Since these titanate layers ought to be separated, the interlayer forces should be as weak as possible. The interlayer force can be reduced by exchanging the potassium ions by protons via an acid exchange reaction. The bulk compound KLTO is therefore treated in nitric acid solution for three days. All potassium and lithium ions are leached out in this process and the HTO phase is formed. Now, protons reside between the layers, and with the inclusion of the cations’ solvatation shells, the distance between the Ti_1.73_O_4_ layers increases, thereby weakening the Coulombic interactions between the titanate layers and the interlayer cations, thus making it easier to exfoliate the layered compound into nanosheets. The exfoliation process itself is triggered by mixing the protonated layered HTO material with TBAOH. Due to their Brønsted acidity, the hydroxyl groups attack the interlayer protons and trigger an acid‐base reaction, forming H_2_O. As the protons are removed from the layers, the negatively charged titanate nanosheets become stabilized by the positively charged TBA^+^ ions. Since TBA^+^ ions are amphiphilic molecules that want to be at the air–water interface, they are able to transfer nanosheets to the air–water interface, where they can be utilized using deposition techniques such as the Langmuir–Blodgett method. The same colloidal suspensions can be used in other deposition techniques as well, like ink‐jet printing. Typically, such suspensions contain about 1–5 g of nanosheets per litre water. The TBAOH/HTO molar ratio influences the concentration of nanosheets in the suspension. A ratio of at least 1:8 is necessary to form a noticeable concentration of nanosheets in the suspension. At higher ratios, up to 2:1, exfoliation happens. At even higher ratios, restacking tends to become dominant. Highly crystalline nanosheets of varying composition, with lateral sizes ranging from hundreds of nanometers to tens of micrometers have been realized with this method. It has been shown that by controlling the grain size of the layered parent compound, the lateral size of nanosheets can be tuned within certain limits.^20^

### Bottom‐up synthesis: self‐assembly of 2D MnO_2_

**Figure 2:**
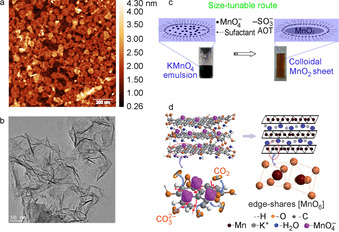
(a) AFM image of MnO_2_ nanosheets deposited on a silicon substrate. (b) TEM image of MnO_2_ nanosheets. (a,b) reproduced with permission.^21^ Copyright 2018, Elsevier. (c) Illustration of size‐tunable route and photo images of MnO_2_ nanosheet colloidal solution. Reproduced with permission from ref. 23 Copyright 2013, Royal Society Chemistry. (d) Schematic of the in situ transformation process. Reproduced with permission from ref. 24 Copyright 2012, Royal Society Chemistry.

In contrast to exfoliation strategies that need high temperatures to synthesize solid state parent compounds, soft‐chemical bottom‐up approaches to prepare MnO_2_ nanosheets under very mild conditions involves chemical oxidation of aqueous Mn^2+^ cations or reduction of Mn^7+^ cations and oxidation of template materials such as graphene oxide. Unilamellar MnO_2_ nanosheets can be prepared by oxidizing MnCl_2_⋅4 H_2_O with H_2_O_2_ in the presence of TBAOH^21^ or tetramethylammonium hydroxide (TMAOH).^22^ Both TBA^+^ and tetramethyl ammonium (TMA^+^) act as charge‐compensating cationic species and soft templates for the formation of MnO_2_ nanosheets. The AFM (Figure 2 a) and TEM (Figure 2 b) images shows the ultrathin structure of MnO_2_ nanosheets with a thickness of around 1 nm.^21^ This value is higher than the crystallographic thickness of monolayer MnO_2_ nanosheets of 0.52 nm, because of the hydration and the presence of organic ions, that is, TBA^+^ or TMA^+^ on the surface of MnO_2_ nanosheets.^21^, ^22^ MnO_2_ nanosheets made by this method have lateral sizes of 50–150 nm, and an average lateral size of 89 nm which makes them usable for inkjet printing.

An alternative chemical reduction process to synthesize size‐tuneable MnO_2_ nanosheets has also been demonstrated.^23^ As shown in Figure 2 c, KMnO_4_ aqueous solution added into iso‐octane was reduced by sodium bis(2‐ethylhexyl) sulfosuccinate (Na(AOT)). The MnO_2_ nanosheet sizes could be tuned by varying the water‐surfactant molar ratio (*W*=[H_2_O]/[AOT]). Their thickness is 2 nm which equals the thickness of three or four monolayers. By increasing the water content, the lateral sizes of nanosheets increased while the thickness was not affected.

The mechanism of another methodology to synthesize MnO_2_ nanosheets using graphene oxide templates is illustrated in Figure 2 d.^24^ First, C−C bonds are broken and C atoms are oxidized to form CO_2_ or CO_3_^2−^. The MnO_4_^−^ is then reduced to [MnO_6_], an octahedral structure. To keep energy stabilization, neighbouring [MnO_6_] octahedral share their edges.^25^ In short, these MnO_2_ nanosheets result from in situ replacement of C atoms of graphene oxide template by edge‐shared [MnO_6_] octahedra through redox reaction.

## Electrical Properties of Nanosheets

For applications in which electronic conductivity plays a crucial role, such as in the supercapacitors that are discussed in section 5.2, the electronic structure of nanosheets, their charge transfer kinetics and the availability of mobile charge carriers are of utmost importance.^26^

Most nanosheet compositions that have been realized so far are oxides of Nb, Ta and/or Ti, and these are typically wide band gap *n*‐type semiconductors (*E*_g_>3 eV). The main contribution to the valence bands are Ti 3*d*‐O 2*p* bonding interactions (or Nb 4*d* or Ta 5*d*), and the conduction bands consist primarily of Ti 3*d* states that are antibonding with O 2*p*.^27^ One of the few exceptions is δ‐MnO_2_, which has a band gap of only 2.23 eV in its pristine (defect‐free) state.^26^

Owing to the anisotropy of nanosheets, it is important to distinguish electron transport in the in‐plane and out‐of‐plane directions. In‐plane electron transport is basically a confined 2D conductivity that is proportional with the charge carrier concentration and carrier mobility within the sheets. On the other hand, electron transport in the out‐of‐plane direction is only possible via surface redox reactions (electron transfer). Furthermore, since all atoms are directly at or very close to the surface, all electronic properties are heavily influenced by the nature and permittivity of the immediate surroundings of the nanosheets. Adsorbed species may thus act as dopants and influence the concentration and/or mobility of charge carriers, and Coulombic charges and local electrical fields that are present within the 2D oxide lattice will be screened by counter ions and the permittivity of the surrounding medium. For example, ionic attachment of C_*n*_H_2*n*+1_‐NH_3_^+^ ions (*n=*14,18) onto Ti_1−*x*_O_2_ nanosheets increased the band gap from 3.84 to 4.06 eV.^28^

Other routes to modulate the conductivity of nanosheets and their charge transfer kinetics are defect engineering and aliovalent doping.^26^ The importance of defect engineering to influence the electronic and redox properties of δ‐MnO_2_ nanosheets has been illustrated clearly in recent studies.^29^ The presence of Mn vacancies in the 2D MnO_2_ lattice leads to new electronic states in the original band gap, thereby reducing the gap effectively from 2.23 to 1.2–1.7 eV, and making the nanosheet more conductive.29b Such defects have also been shown to be very important for improving the specific capacitance of δ‐MnO_2_‐based pseudocapacitors,29a see also section 5. Another way to improve conductivity is to introduce aliovalent substitutional dopants, for example, Co^2+^ and/or Fe^3+^ ions into Ti_1−*x*_O_2_, or Ru into MnO_2_. This leads to additional mobile charge carriers in the lattice, and the formation of new energy states in the band gap.^30^ Both phenomena may facilitate an increase of conductivity.

## Nanosheet‐Based Films and Micropatterns

### Langmuir–Blodgett films

In order to deposit metal oxide nanosheets on solid substrates, several deposition techniques are developed in the decade, such as Langmuir–Blodgett (LB) deposition^31^ and ink‐jet printing.^32^ LB deposition is a monolayer deposition method, which allows detection of the intrinsic properties of densely packed monolayer films of single (layer) nanosheets. Ink‐jet printing is a more mature deposition process to make devices from nanosheet solution. Compared with LB deposition, ink‐jet printing is much faster and it is much easier to vary the thickness of the nanosheet stack, which also makes it suitable for device development. The deposition process, limitations and suitable patterns of LB deposition and ink‐jet printing are discussed in this section and section 4.3, respectively.

Normally, after the exfoliation or bottom‐up synthesis process, metal oxide nanosheets are homogeneously dispersed in the solution. The surfaces of the nanosheets are covered with hydrophobic organic groups, for example, TBA^+^, that are anchored to the sheets via electrostatic interactions. These hydrophobic organic groups will help to transport nanosheets to the liquid–air interface of the solution and form a relatively dense nanosheet monolayer at liquid–air interface.

Langmuir–Blodgett deposition is an effective technique to transfer such Langmuir monolayers from the air‐solution interface to a solid substrate. Langmuir monolayers can be formed by organic molecules,^34^ polymers,^35^ and nanosheets.^11^, ^36^ In this review, the discussion will be limited to using Langmuir–Blodgett deposition for metal oxide nanosheet thin films.

**Figure 3:**
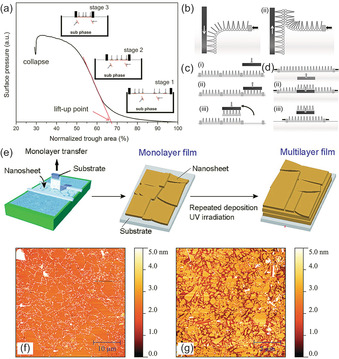
(a) Surface pressure–surface area isotherm of a dilute nanosheet solution and sketch of Langmuir trough in different stages of the isotherm. Reproduced with permission.^19^ Copyright 2016, American Chemical Society; (b) Vertical pull‐up (i) and push‐down (ii) method of LB deposition. (c) Horizontal lifting method of LB deposition. (d) Horizontal pull up method of LB deposition. Reproduced with permission.^31^ Copyright 2013, Wiley‐VCH; (e) Fabrication procedure for multilayer films using the LB method. Reproduced with permission.^33^ Copyright 2010, American Chemical Society. (f) LB‐derived titanate film with 97 % coverage (suspension concentration 30 mg L^−1^, surface pressure 20 mN m^−1^); (g) LB titanate film with 63 % coverage (suspension concentration 20 mg L^−1^, surface pressure 5 mN m^−1^). Reproduced with permission.9d Copyright 2014, American Chemical Society.

LB deposition starts with the formation of a dense nanosheet monolayer, as shown in Figure 3 a (steps 1–3). Firstly, the nanosheet solution is transferred in a shallow trough (Langmuir trough) to allow more nanosheets to move to the liquid‐air interface. Then, the barriers compress the liquid–air interface to increase the nanosheet concentration at the liquid–air interface and form a dense nanosheet monolayer. In this stage, the change of the surface pressure at the liquid–air interface is monitored in situ by a surface balance, which results in a surface pressure–surface area isotherm as shown in Figure 3 a.^19^ The surface pressure usually increases with the decrease of surface area until a plateau is reached, which is taken as evidence of the successful formation of a dense nanosheet monolayer. Yuan et al. showed that the degree of monolayer coverage can be controlled by tuning the surface pressure during the deposition process. However, the bulk concentration of nanosheets in the solution has a much smaller impact on the coverage.9d Thus, amphiphiles are almost always used as surfactants to increase the nanosheet concentration at the liquid–air interface,^37^ although Muramatsu et al. showed that it is also possible to form a dense nanosheet monolayer film without amphiphiles.^38^

The Langmuir monolayer transfer process is shown in Figure 3 b. The vertical pull‐up method i) is used for hydrophilic substrates (such as Si wafer, SiO_2_ etc.) and the vertical push‐down method ii) is used for hydrophobic substrates (see Figure 3 b). The vertical pull‐up method is the most commonly used method. Using a hydrophilic substrate as an example, the substrate is firstly immersed in the solution before the lift‐up process is started. During the deposition process, the surface pressure is kept constant by further compressing the monolayer when needed. In this stage, the transfer ratio, which is defined as the ratio between the decrease of area at the liquid‐air interface and the covered area on the substrate, can be recorded to show the quality and quantity of the Langmuir monolayer. These two vertical methods are massively used for nanosheet layer deposition. Langmuir–Blodgett deposition is not limited to the vertical method. There are several modified versions of this transfer technique, such as the horizontal lifting method, also called the Langmuir–Schaefer method (LS, in Figure 3 c) and the horizontal pull‐up method (in Figure 3 d).^31^ These methods are rarely used to develop nanosheet monolayers, but rather for molecular thin films. The LB process for monolayer and multilayer films is schematically depicted in Figure 3 e. Examples of a densely packed (97 %) monolayer film and a partially covered (63 %) monolayer film of titanate nanosheets are shown in Figure 3 f, g.

### LB multilayer films and heterostructures

Langmuir–Blodgett‐based nanosheet monolayers are usually made to measure the intrinsic properties of single nanosheets or a single layer of nanosheets. Nanosheet multilayers are more relevant for devices. Nanosheet monolayer films usually contain a small percentage of overlapping areas and uncovered gaps. By repeating the LB process several times in a layer‐by‐layer (LbL) fashion, multilayer nanosheet films can be made to prevent such issues. In 2010, Sasaki et al.^33^ reported a high‐k dielectric multilayer Ca_2_Nb_3_O_10_ nanosheet thin film made by such a process. In order to decompose the TBA^+^ ions still present on the surface of the nanosheet after LB deposition, the films were irradiated by UV white light after every deposition step, as illustrated in Figure 3 e. The leakage current decreased upon increase of the numbers of Ca_2_Nb_3_O_10_ (CNO) monolayers in the device structure. Similarly, Kang et al.^39^ reported a CdS thin‐film transistor using a 10‐layer CNO nanosheet‐based thin film as a gate insulator. The same type of CNO nanosheet films are also used in organic light‐emitting devices (OLED).^40^ Yoo et al.^41^ reported Ag‐doped RuO_2_ nanosheet multilayers for flexible transparent electrodes, and noted that the electrical conductivity increased with the number of layers. Similarly, the LbL method can also be applied to fabricate heterostructure thin films, for example, a LaNb_2_O_7_/Ca_2_Nb_3_O_10_ perovskite nanosheet superlattice thin film.^42^ This heterostructure thin film showed superior ferroelectricity and potential for application in nanodevices. The same group also developed a Ti_0.8_Co_0.2_O_2_/Ca_2_Nb_3_O_10_ nanosheet heterostructure thin film using the same method and discovered that ferromagnetism and ferroelectricity coexist in this system.^43^

Next to the LbL approach in combination with LB deposition, the sequential stacking of oppositely charge nanosheets is also an applicable method to synthesize nanosheet heterostructure thin films. Li et al.^44^ reported Ti_0.91_O_2_/Mg_2/3_Al_1/3_(OH)_2_ and Ca_2_Nb_3_O_10_/Mg_2/3_Al_1/3_(OH)_2_ nanosheet heterostructure thin films synthesized in this way. Mg_2/3_Al_1/3_(OH)_2_ is a layered double hydroxide (LDH) nanosheet that carries a positive surface charge in colloidal state, while Ti_0.91_O_2_ and Ca_2_Nb_3_O_10_ metal oxide nanosheets are negatively charged. The process is carried out by immersing a substrate in an LDH nanosheet/formamide solution, followed by immersion in a metal oxide nanosheet/formamide solution, with a cleaning process (immersion in pure formamide) in between. The process can be repeated multiple times. Unfortunately, only a limited number of cationic nanosheet compositions is available, which limits the method to a small range of layered nanocomposite films. The method is not able to fabricate fully covered layers, that is, it does not provide the same degree of film quality as LB deposition does. The method is nowadays mostly used for synthesizing superlattice micro/nanocrystals.^45^ It would be desirable to develop this method for heterostructure nanosheet thin films further, because combinations of different layers may even show emergent properties that cannot be found in the individual layers.^46^

### Inkjet printing of nanosheets

**Figure 4:**
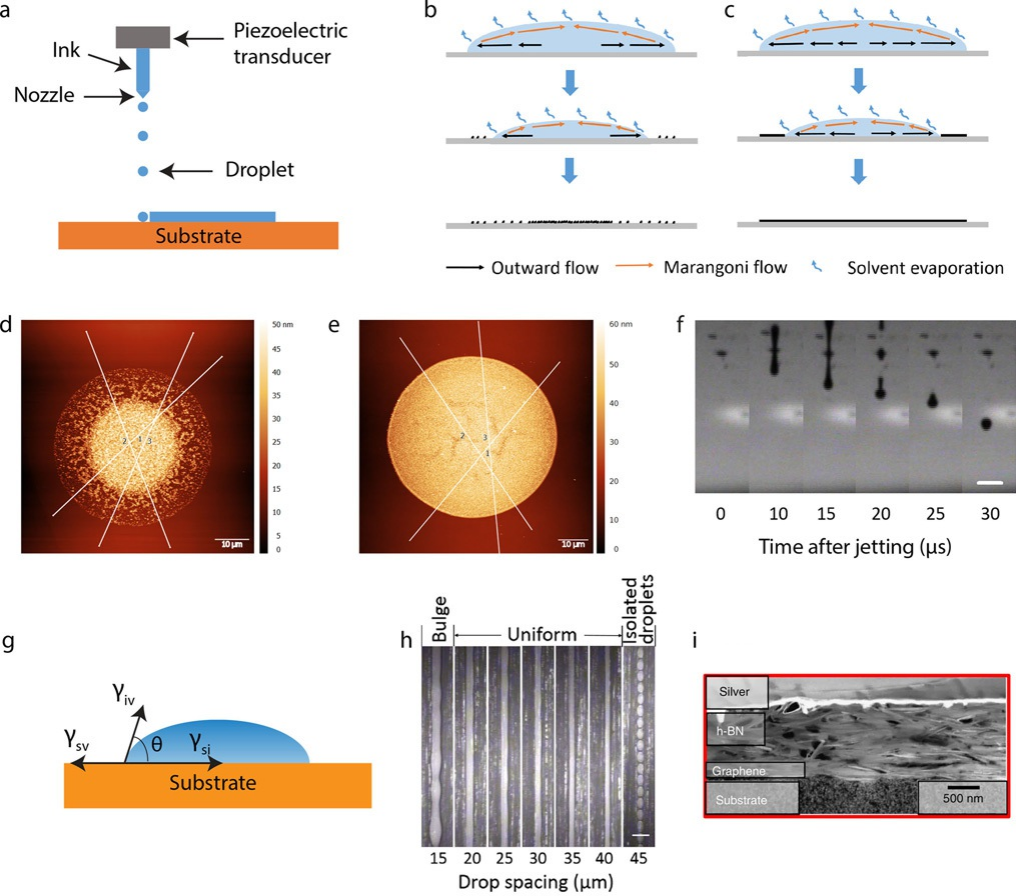
(a) Schematic of drop‐on‐demand piezoelectric type inkjet printer. Droplet drying process with (b) excess and (c) optimized surfactant concentration. AFM image of a printed single droplet on glass substrate with (d) excess and (e) optimized surfactant concentration. (f) Optical images of MnO_2_ ink droplet formation vs. time. (b–f) Reproduced from ref. 21. (g) Schematic of the wetting of a droplet on substrate. (h) Optical images of printed lines with varying droplet spacing. The scale bar is 100 μm.^21^ (i) High angle annular dark‐field‐scanning transmission electron microscopy cross‐sectional of printed heterostructures. Reproduced from ref. 51.

As a digital, non‐contact and high‐resolution deposition technique, inkjet printing attracted great attention in the field of flexible electronics.^47^ Due to the mask‐free feature of inkjet printing, inks can be deposited onto various substrates, such as rigid substrates, paper and flexible polymer substrates. The two main inkjet printing modes are continuous inkjet and drop‐on‐demand inkjet printing. Drop‐on‐demand piezoelectric type inkjet printers have been widely used for lab research. To jet the ink from the nozzle, an electric field is applied to the piezoelectric material in order to create a force that pushes the ink through the nozzle onto the substrate (Figure 4 a).

The crucial issues for successful inkjet printing are ink formulation and optimization of printed patterns. In order to form high quality patterns, droplet formation needs to be optimized to prevent undesired satellite droplets or tails. It is possible to optimize droplet formation by optimizing the firing waveform or the voltage of the nozzles of the inkjet printer. Lee et al. reported that double waveforms with two square pulses could overcome the problems associated with the use of low viscosity inks in inkjet printing.^48^ Inks for inkjet printing typically consist of small entities dispersed in solvents to form homogeneous colloidal suspensions, optionally also containing certain additives. A high concentration of solids and high ink stability are the two main issues for 2D material‐based preparation of inks. Additives like surfactants can be used to improve the dispersion of 2D materials in the inks. Solvents play an important role in the inkjet printing process. Various solvents like water and organic solvents are often used for inkjet printing. However, water is not an ideal solvent for inkjet printing due to its high surface tension (around 70 mN m^−1^) and low viscosity (around 1 mPa s).^21^ To evaluate ink printability, the inverse Ohnesorge number *Z* which contains the physical properties of functional ink, is defined as [Eq. (1)]:

(1) $$Z=\left(\alpha \rho \gamma \right){}^{1/2}/\eta$$

in which *α* is the nozzle diameter, *ρ* is the density, *γ* is the surface tension, and *η* is the viscosity of the ink. To prepare printable inks, the lateral dimensions of functional materials dispersed in the ink should be less than 1/20 of the nozzle diameter to avoid nozzle blocking during printing.^49^ The ink viscosity should also be optimized to meet the requirements of the printer. The ideal viscosity range for inkjet printing is between 2–25 mPa s.^50^ The surface tension of the ink also plays an important role in ink formulation engineering. Low surface tensions lead to spontaneous dripping of droplets while high surface tensions make it difficult to print.

Our group reported the printable MnO_2_ nanosheets ink by adding additive to modify the fluidic properties. Triton X‐100 was used to not only decrease the surface tension of water to 46 mn m^−1^ but also to keep the electrostatic stabilization of MnO_2_ nanosheets. Propylene glycol was added to increase the viscosity of water to 1.71 mPa s. The concentration of Triton X‐100 must be carefully optimized to get a high‐quality printed film. As shown in Figure 4 b, excess Triton X‐100 can lead to non‐uniform distribution of MnO_2_ nanosheets, as shown by the atomic force microscopy (AFM) image in Figure 4 d. An optimized Triton X‐100 concentration led to uniform deposition of MnO_2_ nanosheets (Figure 4 c), as illustrated by the AFM image in Figure 4 e. The optimized Triton X‐100 concentration was able to balance the outflow force and weak Marangoni flow, thereby reducing the “coffee‐ring” effect.^52^ The *Z*‐value based on above values is about 19 for the water‐based MnO_2_ nanosheets ink. The optimal *Z*‐value suggested by Jang et al. is between 4 and 14.^53^ However, our finding shows that ink with *Z‐*value outside of optimal range can also be printed smoothly (Figure 4 f).

Interface engineering is another key issue for high quality inkjet printing. The wetting process is defined by Young's equation [Eq. (2)]:

(2) $$\gamma {}_{\mathrm{sv}}=\gamma {}_{\mathrm{si}}+\gamma {}_{\mathrm{iv}}\;\cos\;\theta$$

in which *γ*_sv_ is the solid (s)–vapor (v) surface energy, *γ*_si_ the solid–liquid (i) surface energy and *γ*_iv_ liquid–vapor surface energy. The *θ* is the contact angle (Figure 4 g). To print continuous lines or films, the contact angle *θ* should be less than 90°, indicating good wetting of printed patterns. Large *θ* (>90°) means poor wetting, resulting in discontinuous printed patterns. Pre‐treatment of substrates by plasma is a common strategy to control the surface energy of the substrates. Tuning the droplet spacing is also an effective method to print continuous patterns. As shown in Figure 4 h, the printed line became bulged with 15 μm droplet spacing because the droplets overlapped significantly with each other. The lines became uniform when the droplet spacing was in the range of 20 to 40 μm. Further increasing the droplet spacing would result in discontinuous lines since the droplets were too far apart to merge with each other.^21^

The interface plays an important role in the performance of devices. It's challenging to achieve sharp and controllable interfaces between printed complex heterostructures, due to the remixing of different 2D materials at the interface. However, a binder can be employed to modify the formulation and to minimize re‐dispersion of nanosheets. Recently, Casiraghi et al. demonstrated that water‐soluble and environmentally friendly polysaccharide xanthan gum binder successfully changed the chemistry of printed 2D crystal inks to allow fabrication of controlled interfaces in all‐printed heterostructures.^54^ The underlying principle is that the viscosity of the ink increases after printing, resulting in minimized remixing of different 2D inks. To keep the 2D crystal ink's electrical properties, a small amount of binder that could have a large rheological effect on the ink was needed. Formulation engineering can also be used to control the interface of printed heterostructures. Torrisi et al. reported that low boiling point solvents (≤100 °C) with fast evaporation at room temperature could reduce the materials transport and re‐dispersion of materials at the heterostructures interface (Figure 4 i).^51^

## Applications of Oxide Nanosheets

### Nanosheets as templates for epitaxial thin film growth

Oxide‐based thin‐film technology is the key to fabricating modern devices with practical applications, such as transistors, solar cells and energy storage. In order to fully utilize devices functions, epitaxial growth plays an essential role to precisely control oxide thin films’ crystal quality in the early stage of fabrication.

However, the integration of oxide‐based devices with the current Si‐based technology has been challenging. The constraint of lattice‐matching on Si or native amorphous SiO_2_ limits the number of oxides that can be grown on Si and the ability to control the properties of their thin films. Moreover, even for non‐Si based technology, the commercialization of oxide‐based devices has been hampered by the high cost and the limited size of single crystal substrates. Low cost and ubiquitous availability of large area glass and plastic satisfy industrial requirements. However, these are usually amorphous substrates, and the fabricated oxide thin films grown onto them are polycrystalline with many orientations and a high concentration of grain boundaries. As a result, they exhibit poor physical properties and thus, devices made from them show poor performance.

In order to realize epitaxial growth of oxides on Si or amorphous substrates, the introduction of a buffer layer is necessary. Oxide nanosheet films are one of the candidates to bridge the gap between substrates and epitaxially grown functional oxide thin films. They have a single well‐defined type of surface termination and the 2D unit cell parameters of many nanosheets are in close agreement with the lattice parameters of a wide range of functional oxides, for example, perovskite‐type oxides. Recently, various oxide films have been epitaxially grown on nanosheet buffer layers on Si and amorphous substrates.^55^ Nanosheets may be considered as micro‐single crystals, with a large library of crystal lattices and symmetric two‐dimensional crystal structures. The crystal orientation of thin films can control their transport, magnetic, and ferroelectric properties. For instance, in the case of La_0.7_Sr_0.33_MnO_3_ films with thickness below 12 nm on LaAlO_3_, the film was insulating with (001) orientation, while it behaved like a metal with (110) orientation.^56^ The use of oxide nanosheets as seed layers may provide new possibilities to control growth orientation, and thus, to tune the properties of functional oxide films. The use of nanosheet seed layers also enables the free choice of substrate, which can be exploited both for practical applications and in fundamental studies.

Epitaxial growth via lattice matching can be realized under strict conditions in terms of structural similarities between a grown oxide crystal and a single crystal substrate. However, dangling bonds are always present on the top‐most layer of the substrate and these restrict the mobility of adatoms at the beginning of epitaxial growth due to their anisotropic nature. This causes the self‐organization of adatoms into a crystal lattice not being favourable in terms of energy. As a result, epitaxy is possible when there are small differences between a grown layer and a single crystal substrate in term of thermal expansion coefficients, crystal symmetry and lattice parameters along a specific orientation. Therefore, coherent epitaxial growth on a single crystal substrate requires a lattice mismatch of less than 8 %.^57^

Van der Waals epitaxy was proposed to lessen the lattice mismatch prerequisite by utilizing cleaved surfaces of substrates, such as mica, metal dichalcogenides, and so on.^58^ These materials have clean surfaces without dangling bonds, on which epitaxial growth of thin films proceeds by exploiting relatively weak and isotropic van der Waals forces. Good epitaxial growth is still possible with a lattice mismatch as large as 20 %.58a

Even for large lattice mismatching between a thin film and a substrate, Narayan and Larson suggested that thin film epitaxy on a single crystal substrate is still feasible via domain matching.^57^ AlN film over (111)Si substrate orientation has a lattice mismatch of about 19 %. Via domain matching epitaxy, a mismatch between 5*×*AlN(2‐1‐10) planes and 4*×*Si(220) planes of less than 1 % still makes it possible for AlN to grow epitaxially on Si(111).^57^ However, it is energetically favourable for the film to have dislocations above a critical thickness, which depends on the type of material grown, and consequently, the grown film may have a high density of dislocation and other defects.

**Figure 5:**
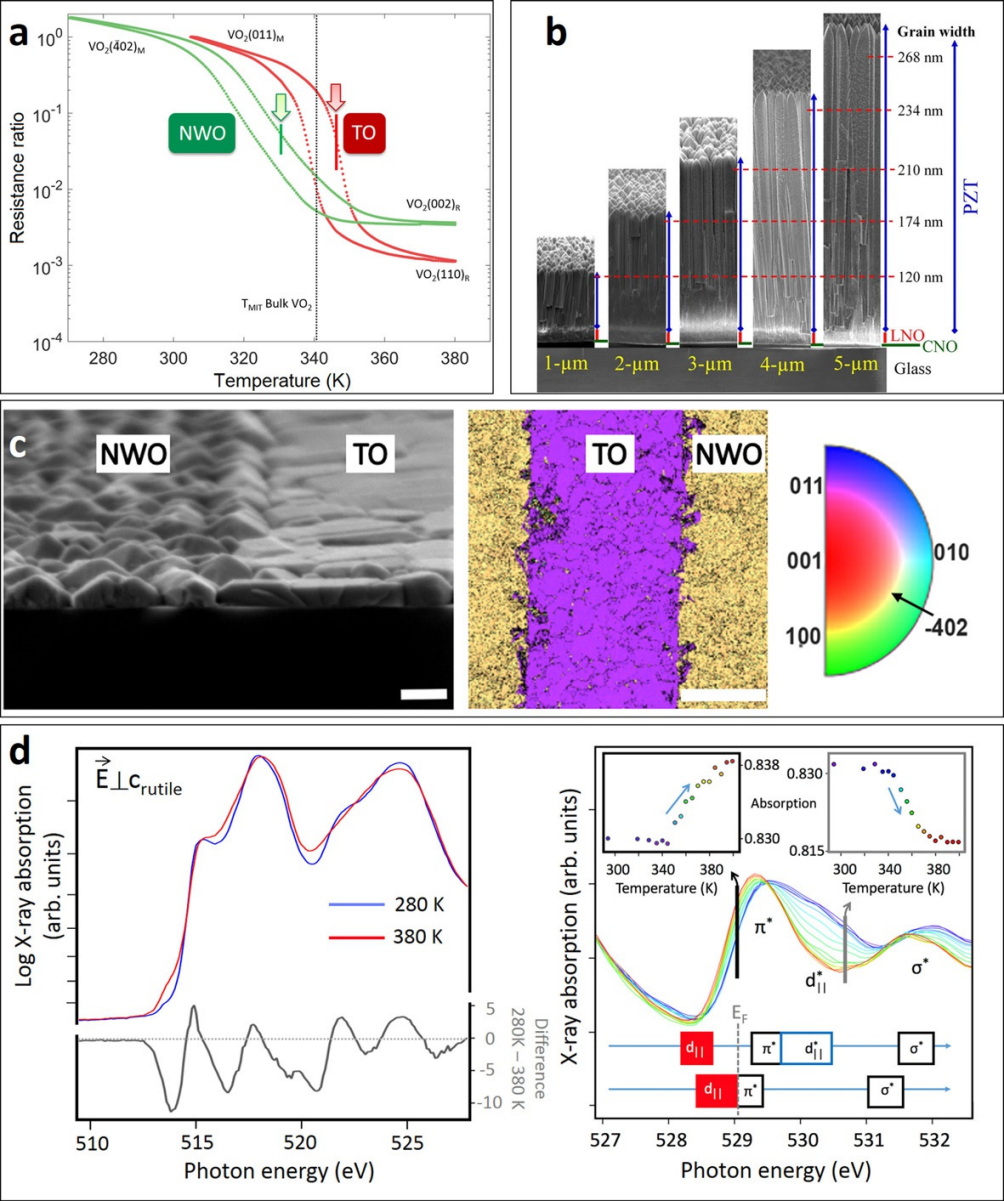
(a) Transport measurements across the metal‐insulator transition of VO_2_ films on TO and NWO nanosheets. (b) Cross‐sectional SEM images of PZT films with different thicknesses on LaNiO_3_/CNO/glass. Reproduced from ref. 60. (c) Cross‐sectional SEM images at the line boundary of VO_2_ film on NWO and TO nanosheets (left), and the inverse pole Figure map, obtained by electron backscatter diffraction, showing the orientation of VO_2_ film in the out‐of‐plane direction (middle) with colour coding (right). (d) Soft X‐ray absorption in transmission: V‐L2,3 of VO_2_ on nanosheets shows clear differences in the spectrum due to the change in electronic structure across the metal‐insulator transition temperature (left), and the O–K edge is recorded as a function of temperature (right), showing the temperature dependence of the two main absorption features of the metal‐insulator transition in the insets. (a), (c), and (d) are reproduced from ref. 55e.

The well‐defined surface structure of nanosheets is quite similar to those of mica and metal dichalcogenides, and nanosheet films can thus be utilized as a seed layer to grow oriented functional oxide thin films. However, it is hard to say if the epitaxial growth on individual oxide nanosheets is “pure” van der Waals or lattice/domain matching epitaxy. Firstly, a localized high charge density compared with van der Waals materials is expected in oxide nanosheets. This may influence adatoms’ interactions in the early stages of growth. Secondly, in van der Waals epitaxy, the grown films have similar lattice constants as the bulk form even when the films are only one unit cell thick.58a There is evidence that anatase TiO_2_, as the grown film, and Ca_2_Nb_3_O_10_^−^ (CNO) nanosheet, as the buffer layer, have lattice constants in restraint with each.^59^ Recently, our report on VO_2_ films grown by pulsed laser deposition (PLD) on Ti_0.87_O_2_^δ−^ (TO) and NbWO_6_^−^ (NWO) nanosheets showed that the metal–insulator transition temperature of VO_2_ films on nanosheets was shifted from its bulk value, which may be the result of the difference in *c*‐axis lattice constant between the nanosheet‐buffered films and the bulk VO_2_ rutile phase (Figure 5 a).55e Even though the lattice or domain matching can be dominant in such epitaxial growth on oxide nanosheets, further study is necessary to verify the growth mechanism on oxide nanosheets.

Although SrRuO_3_ (SRO) is generally ferromagnetic around 160 K, it is one of the most important conducting perovskites because of its common application as an electrode in oxide heterostructures at room temperature.^61^ Controlling the properties of SRO on Si substrates is a good illustration of the ability of integration of oxide based devices with Si based technology. Our group has shown that SRO grows epitaxially in (001)_pc_ and (011)_pc_ orientations on CNO and TO nanosheets, respectively, for which “pc” refers to a pseudocubic crystal symmetry.9c, 55c However, direct growth of SRO on nanosheets results in minor orientations of (011)_pc_ and (001)_pc_ on CNO and TO.9c The introduction of a few unit cells of SrTiO_3_ prior to the deposition of SRO completely suppresses these minor orientations on these two types of nanosheets.55c Even though the SRO films have grain boundaries and random in‐plane orientations due to the micrometer dimensions and random distribution of oxide nanosheets on Si, their resistivity is comparable with those on single crystal STO substrates.55c In addition, the magnetic easy axis (“easy” refers to the direction within a crystal structure in which magnetization can be altered most easily) of SRO, which can be parallel or perpendicular to the surface of the film, can be easily tuned by choosing properly buffered CNO or TO nanosheets,55c independently from Si substrate.

Pb_1−*x*_RE_*x*_(Zr_*y*_Ti_1−*y*_)O_3_ materials, with RE representing a rare earth element, are one of the most important perovskite family of ferroelectrics, relaxor ferroelectrics, antiferroelectrics and piezoelectrics for various applications, such as microelectromechanical systems and energy storage devices.55d, 60, 62 The deposition of this family of materials on Si substrates is the next step in realizing oxide‐based devices. Ferroelectric PbZr_0.52_Ti_0.48_O_3_ (PZT), which is known to have the largest dielectric constant and piezoelectric coefficients in its family, was deposited on oxide nanosheets on Si with SRO as electrodes.55d PZT films have main (001) and (011) orientations, the same as SRO films on CNO and TO nanosheets, respectively, but minor orientations are present in PZT films (011) on CNO and (001) on TO nanosheets as well.55d They can be suppressed by introducing an STO layer before the deposition of the whole stack of SRO/PZT/SRO. It is worth mentioning that (001) oriented PZT films grown on CNO nanosheets on an amorphous (glass) substrate demonstrates the best piezoelectric coefficient (490 pm V^−1^) in piezoelectric films, thanks to columnar microstructure and strong orientation of the films along the *c*‐axis (Figure 5 b).^60^ In addition to PZT composition, relaxor ferroelectric Pb_0.9_La_0.1_Zr_0.52_Ti_0.48_O_3_ and antiferroelectric PbZrO_3_ have also been drawing attention for energy storage devices.62b, 62c, 62d The films of these two compositions can be oriented in the same manner as PZT films by using oxide nanosheets. The performance of these devices scales with the recoverable energy storage density (*U*_reco_), which depends on the critical electric breakdown field (*E*_BD_), and the maximum and remanent polarizations. Different kinds of nanosheets can be used to tune the microstructure of films. While Pb_0.9_La_0.1_Zr_0.52_Ti_0.48_O_3_ film has a dense microstructure on CNO nanosheets, it has a columnar one on TO nanosheets.62c As a result, *E*_BD_ increases for the dense film, leading to a higher *U*_reco_ value.62c

Vanadium dioxide VO_2_ is one of the leading candidates for electronic oxide devices for low power operation in field‐effect type or neuromorphic electronic devices.^63^ VO_2_ films are (001)_R_ and (110)_R_ out‐of‐plane oriented in the high temperature rutile (R) phase on NWO and TO nanosheets, respectively.55e As we mentioned above, the former film has a metal‐insulator transition temperature (*T*_MIT_) at 332 K, lower than the bulk value of 341 K, whereas the latter has *T*_MIT_ at 347 K, higher than the bulk value.55e It is known that the compressive strain on the rutile *c*‐axis of VO_2_ leads to a decrease of *T*_MIT_, while the tensile strain on it increases *T*_MIT_.^64^ We suggested that the same phenomena happen to VO_2_ films on NWO and TO nanosheets.55e

In order to highlight the additive potential to the free choice of substrates and demonstrate how well oxide nanosheets can be controlled on the micrometer‐scale, our group showed that two different orientations of functional oxides can be realized on a single substrate in a defined pattern by utilizing lithography.55c, 55e For example, the alternative line pattern between (−402)_M1_ and (011)_M1_ (where M1 refers to the monoclinic phase) VO_2_ is achieved by growing on the same pattern of NWO and TO nanosheets (Figure 5 c).55e Although the minimum feature size of the patterns may be limited by the size of nanosheets, either top‐down etching of nanosheets, or size‐controlling nanosheets before or after exfoliation is feasible to achieve smaller feature sizes. The ability to micropattern the orientations of functional oxides on the micrometer‐scale may open new functionalities for devices that single crystal substrate cannot provide. Next to micrometer‐scale patterning, a methodology to transfer pre‐grown nanosheet‐seeded thin films from a sacrificial mica substrate to any arbitrary substrate, for example, thermally sensitive flexible plastic substrates, has also been developed.^65^

In addition to potential applications, oxide nanosheets can facilitate the fundamental study of various materials, in which mechanisms or structural changes usually happen along a specific crystal axis. Advanced characterization techniques, such as soft X‐ray absorption spectroscopy in transmission mode and transmission electron microscopy (TEM), also need the film thickness to be in the range to facilitate electron or X‐ray transparency. Both requirements are hardly achieved on single crystal substrates or amorphous X‐ray transparent TEM grids at the same time. Remarkably, oxide nanosheets are transparent to electrons and X‐rays thanks to their thickness of only a few atomic layers. We gave a proof of principle for the case of VO_2_ in soft X‐ray absorption spectroscopy (Figure 5 d).55e

### Nanosheets in supercapacitors and batteries

**Figure 6:**
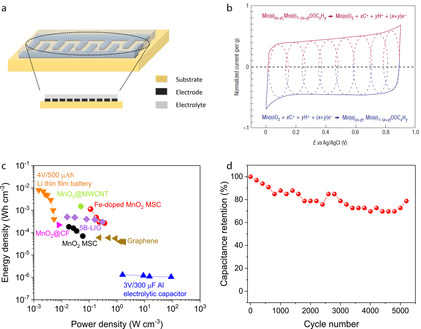
(a) Schematic of in‐plane interdigitated configuration MSC. (b) Cyclic voltammetry of MnO_2_ electrode in 0.1 m K_2_SO_4_ aqueous electrolyte. Reproduced with permission from ref. 76 Copyright 2008, Springer Nature. (c) Ragone plot of printed Fe‐doped MnO_2_ nanosheets MSC and recent data from literature. (d) Cycling stability of Fe‐doped MnO_2_ nanosheets MSC at a current density of 70 μA cm^−2^. (c,d) Reproduced from ref. 77.

Due to their unique atomically thin structure and large surface area, 2D nanosheets have recently attracted considerable attention for their potential application in the field of energy storage.^11^, ^66^ Micro‐supercapacitors (MSCs) which have interdigitated electrode structures show great potential for integration with chips or flexible electronics.^67^ As shown in Figure 6 a, the in‐plane interdigitated configuration of MSCs using stacked nanosheet electrodes in the same plane, offers fast ionic movement in horizontal directions and an increase of the accessibility of the electrodes. Inkjet printing, a digital printing technique, has been widely used for the fabrication of in‐plane interdigitated MSCs.^68^ Graphene, which was exfoliated in 2004, shows promise as electrode materials for supercapacitors because of its high electrical conductivity, excellent stability and mechanical flexibility and large specific surface area.^69^ Li et al. reported scalable fabrication of, fully inkjet‐printed graphene MSCs.^70^ Graphene was printed as electrode on flexible polyimide substrate followed by printing poly(4‐styrenesulfonic acid) as electrolyte on top of electrodes. These fully printed MSCs exhibited areal capacitances of around 0.7 mF cm^−2^. Furthermore, large scale MSC arrays which were printed on both silicon wafers and polyimide can be charged to voltages as high as 12 V. Beyond graphene, a wide range of other novel 2D nanosheets have been proposed to serve as electrode materials for supercapacitors, such as MoS_2_,^71^ black phosphorus,^72^ MXene^73^ and transition metal oxides.^11^, ^74^ Recently, Zhang et al. reported the additive‐free MXene ink for printed MSCs.^75^ To fabricate all‐MXene MSCs, MXene ink was first printed on substrates followed by coating with H_2_SO_4_‐poly (vinyl alcohol, PVA) as a gel electrolyte on top of the device. The printed MSCs exhibited volumetric capacitances up to 562 F cm^−3^ and energy densities of 0.32 μWh cm^−2^.

MnO_2_ nanosheets, which exhibit a large theoretical capacitance (1233 F g^−1^), have been reported as electrodes for printed supercapacitors.^32^, ^74^ However, the electronic conductivity of MnO_2_ is low (10^−5^ to 10^−6^ S cm^−1^ for Na‐birnessite).^78^ One strategy to improve the electronic conductivity of MnO_2_ is by defect engineering.^79^ An alternative strategy is to combine MnO_2_ with highly electron conductive materials such as graphene.^80^ The charge storage mechanism of MnO_2_ nanosheets occurs by the reduction of Mn^4+^ to Mn^3+^, e.g.:

$$\mathrm{MnO}{}_{2}+xC{}^{+}+xe{}^{-}\to \mathrm{MnOOC}{}_{x}$$

in which A is an alkali ion or a proton. As shown in Figure 6 b, MnO_2_ electrode show fast, reversible surface redox reactions leading to pseudocapacitive charge storage in mild aqueous electrolytes.^76^ The cyclic voltammograms of MnO_2_ electrode in Figure 6 b is close to electrochemical double layer capacity.

Our group demonstrated all‐solid‐state MSCs by inkjet printing MnO_2_ nanosheets as active materials.^21^ MnO_2_ nanosheets were synthesized following the bottom‐up synthesis strategy outlined in section 2.2. To prepare water‐based printable MnO_2_ inks, Triton X‐100 and propylene glycol were added into the MnO_2_ solution to modify the surface tension and viscosity of the solution, respectively.^54^ By formulation engineering of MnO_2_ nanosheets ink, it can be printed on arbitrary substrates without the “coffee‐ring” effect. To fabricate asymmetrical MSC on flexible polyimide substrate, MnO_2_ nanosheets ink was firstly printed on polyimide with in‐plane interdigitated configuration followed by thermal annealing. Highly conductive poly(3,4‐ethylenedioxythiophene): poly(styrenesulfonate) (PEDOT: PSS) ink was printed on top of MnO_2_ to serve as current collectors. To complete the solid state MSC device fabrication process, PVA/LiCl as gel electrolyte was dropped on top of the device and dried at room temperature. The MSC exhibited high volumetric capacitances of 2.4 F cm^−3^ and high energy densities of 1.8×10^−4^ Wh cm^−3^ at power densities of 0.018 Wcm^−3^ (Figure 6 c). To improve the electrochemical performance of MnO_2_ micro‐supercapacitor, 5 % substitutional iron doping was applied and the resulting Fe‐doped MnO_2_ nanosheets were inkjet‐printed as active materials with interdigitated structures for MSC.^77^ Cobalt and nickel were also active dopants but to a much lesser extent than Fe. The Fe‐doped MnO_2_ MSC exhibited a volumetric energy density of 1.13×10^−3^ Wh cm^−3^ at volumetric power density of 0.11 Wcm^−3^, both more than six times higher than the MSC based on undoped MnO_2_ nanosheets (Figure 6 c). Furthermore, the Fe‐doped MnO_2_ MSC showed good mechanical flexibility and cycling stability with a capacitance retention of 78.7 % after 5200 cycles of charge/discharge (Figure 6 d). These MnO_2_ based printed MSCs show promise as energy storage units for utilization in flexible electronics. These performance data confirm that inkjet printing is a promising method for manufacturing flexible energy storage devices like supercapacitors and batteries.

Nanosheets have also gathered interest for utilization in lithium ion batteries. When using nanosheets as a solid‐state electrolyte, batteries can be made thinner. Usage of 2D materials leads to shorter pathways for ions, increasing the rate capacity of the battery. Moreover, less packaging compared to liquid electrolytes is needed due to the absence of possible leakages, which makes that these types of batteries are intrinsically safer. The lifetime of solid‐state electrolytes is superior to liquid electrolytes, because they do not degrade easily compared to using liquid electrolytes. The ionic conductivity, however, is thus far lower than in liquid electrolytes. Different 2D materials have already been explored for the different components of a battery. For example, graphene is a commonly used anode material for lithium ion batteries, because of its capability for reversible lithium ion intercalation in the layered crystals. The structural similarities of graphene nanosheets to graphite may provide another type of intercalation anode compound.^81^ MXenes have also been attracting a great deal of attention as emerging low‐cost and high energy‐density anodes for batteries, not only for lithium, but also for non‐lithium batteries.^82^ LiMPO_4_ (M=Fe, Mn, Co, Ni) has become of great interest as cathodes for next‐generation high‐power lithium ion batteries. This is an olivine‐type 2D material, which can be optimized by a solvothermal lithiation process to increase the lithium diffusion.^83^ And leaf‐like V_2_O_5_ nanosheets can be fabricated via a facile green approach as high energy cathode material.^84^ Since nanosheets have a large surface to volume ratio, it is possible to attach functional groups on the nanosheet surface or dope the 2D sheets with foreign elements. For example, titanate nanosheets have been used as electrode in batteries, but their electronic conductivity is rather low. By doping titanate with an element like niobium,30a the conductivity may be increased to facilitate its use as an electrode.

### Catalyst design using nanosheets as templates

The concept of employing nanosheets as templates to control the oriented growth of PLD‐derived films can also be applied to wet‐chemical deposition processes. The deposition and crystallization steps are typically separated in a wet‐chemical process: first, an amorphous precursor phase is deposited onto a nanosheet. In the subsequent step the precursor is thermally annealed and crystallized. Sol–gel‐derived anatase films have been grown from (NH_4_)_2_TiF_6_ and H_3_BO_3_ on Ca_2_Nb_3_O_10_ and Ti_0.87_O_2_ and it was shown that the nanosheets could survive both the conditions during the wet‐chemical deposition process, and the subsequent thermal annealing step at 450 °C.^85^ It was found that {100} and a few {001} facets were exposed at the surface of the anatase film grown on Ti_0.87_O_2_. The anatase film grown on Ca_2_Nb_3_O_10_ exposed mainly its {001} facets. The differences between the two forms of anatase became prominent in photocatalytic hydrogen formation experiments involving these films.^85^

### Nanosheet‐based soft hybrid materials

**Figure 7:**
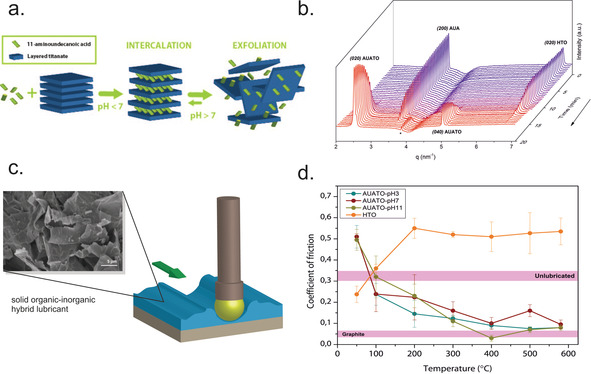
(a) Schematic of intercalation of AUA into HTO by ion exchange at pH < IEP, followed by exfoliation at pH > IEP. (b) Time‐resolved small‐angle X‐ray scattering profiles of the reaction between HTO and AUA to form AUATO nanocomposite. Scattering peaks of AUA and HTO disappear within minutes while the AUATO signal emerges. (a,b) Reprinted with permission.^86^ Copyright 2017, Wiley‐VCH. (c) Schematic of pin‐on‐disc friction experiments and a SEM image displaying hexylamine‐intercalated titanate nanosheet powders used as solid lubricant. Reprinted with permission from ref. 12. Copyright 2016. American Chemical Society. (d) Coefficient of friction (CoF) of AUATO films obtained at varying pH and different temperatures. The reference CoFs of unlubricated contacts (0.32) and graphite (0.050) are also displayed. Reprinted with permission.^86^ Copyright 2017, Wiley‐VCH.

Although the emphasis in this review is on the synthesis and applications of the 2‐dimensional forms of the oxides, the parent layered oxides may also be chemically modified with organic intercalants to render a layered hybrid material. Such a material is relatively easy to deform under shear force, and might thus find application where that property is exploited, for example, as a solid lubricant.^12^, ^86^ *n*‐Alkylamines, with alkyl chains having 3 to 12 carbon atoms, can easily intercalate into protonated layered oxides, such as H_1.07_Ti_1.73_O_4_ (HTO).^12^ The intercalation process is driven by the acid‐base reaction between the intercalating amines and the interlayer protons. The nanosheet layer distance in the hybrid can be controlled by the length of the *n*‐alkylamine chain and the amine/HTO ratio. In another study, the zwitterionic amino acid 11‐aminoundecanoic acid (AUA) was intercalated into HTO through ion exchange, see Figure 7 a and 7b.^86^ The amino acid molecules formed an ordered paraffinic bilayer in the gallery region of the layered host and replaced H^+^ at pH below the isoelectric point (IEP) of AUA (pH 7.85). The nanocomposite can be exfoliated into a disordered structure at pH≫IEP, where the amino acid becomes negatively charged and destabilizes the ordered layered structure. Above 180–200 °C, the amino acid polymerized into nylon‐11 confined between crystalline titanate monolayers. In both studies, the intercalation process followed first‐order Langmuir‐type kinetics, that is, random irreversible adsorption with an adsorption rate proportional to the number of available adsorption sites. Both hybrids showed good performance as high temperature solid lubricants (Figure 7 c), showing friction coefficients similar to that of the state‐of‐the‐art material graphite (Figure 7 d).

## Conclusions and Outlook

A diverse range of nanosheet compositions derived from layered metal oxides has been reported. Most nanosheets are made via soft chemical exfoliation routes, but a few compositions, such as δ‐MnO_2,_ can also be synthesized directly by a solution process involving self‐assembly of molecular precursors in water. The composition and doping levels in nanosheets can be modulated relatively easily via modification of the composition and grain size of the layered parent compounds. This versatile and generic approach provides various easy handles to introduce and optimize the functional properties and dimensions of oxide nanosheets. As is illustrated in this review, by utilizing 2D nanosheets as elementary building blocks for further materials design, a wide range of functional layered hybrid nanocomposites, nanosheet‐based monolayers and micropatterned films with diverse properties can be generated for emerging applications in energy conversion and storage, (nano)electronics, catalysis and even mechanical engineering.

## Conflict of interest

The authors declare no conflict of interest.

## Biographical Information

*Melvin Timmerman received his Bachelor and Master's degree in chemical engineering at the University of Twente*, *the Netherlands. He is currently a PhD candidate in the MESA+ Institute for Nanotechnology at the University of Twente. Under the supervision of Prof. J.E. ten Elshof and Prof. M. Huijben, he is working on two‐dimensional materials for energy storage applications*.

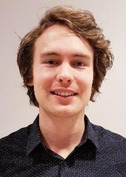

## Biographical Information

*Rui Xia received his Bachelor's degree from Wuhan University of Technology under the supervision of Prof. Liqiang Mai and Master's degree from University of Twente under the supervision of Prof. Johan E. ten Elshof. He is currently a PhD candidate focusing on the ion diffusion process in energy storage device and micro‐batteries supervised by Prof. Johan E. ten Elshof and Prof. Mark Huijben*.

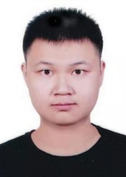

## Biographical Information

*Phu Tran Phong Le received his Bachelor's degree in Chemical Engineering from Ho Chi Minh City University of Technology, Vietnam, and Master's degree in Advanced Materials from University of Groningen, the Netherlands. He is currently a PhD candidate in the Inorganic Materials Science group at University of Twente, the Netherlands. His current research interest focuses on electronic properties of functional oxides grown on oxide nanosheets*.

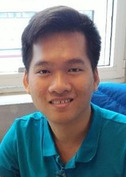

## Biographical Information

*Yang Wang is a PhD candidate under Dr. Professor J. E. ten Elshof's supervision in the MESA+ Institute for Nanotechnology at the University of Twente, the Netherlands. He is currently focusing on 2D materials and printing 2D materials for flexible energy storage devices*.

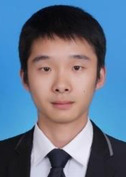

## Biographical Information

*Dr. Johan E. ten Elshof is professor of Inorganic and Hybrid Nanomaterials Chemistry at the MESA+ Institute for Nanotechnology of the University of Twente in Enschede, Netherlands. His research focuses on novel functional metal oxide and organic‐inorganic nanomaterials, nanopatterns and nanostructures, with specific emphasis on low‐dimensional structures like flexible nanofibers, nanosheets and nanowires. Main application areas of these materials are in the areas of energy materials and nanoelectronics*.